# Supplementary material for: Relationship between the Rate of Perceived Stability, electrodermal activity and task performance during balance challenges in chronic stroke
Source: PLoS One. 2026 Jan 8;21(1):e0322825. doi: 10.1371/journal.pone.0322825 (PMC12782383; doi:10.1371/journal.pone.0322825)
Supplement: Attachment — Submitted filename: Response to Reviewer 2_Aug21_Clean.docx [file pone.0322825.s002.docx]

**Answers to Reviewers**

Journal Requirements

1. Please ensure that your manuscript meets PLOS ONE's style requirements, including those for file naming. The PLOS ONE style templates can be found at

[https://journals.plos.org/plosone/s/file?id=wjVg/PLOSOne_formatting_sample_main_body.pdf](https://track.editorialmanager.com/CL0/https:%2F%2Fjournals.plos.org%2Fplosone%2Fs%2Ffile%3Fid=wjVg%2FPLOSOne_formatting_sample_main_body.pdf/1/010f0197f438383e-8fd3286f-ab0c-4e96-b630-e1a1da2f4836-000000/WL6-9G4snzs8wvnRkTvy0-sIdB8Jtz7LamCcuE8Gmz0=219)  and

[https://journals.plos.org/plosone/s/file?id=ba62/PLOSOne_formatting_sample_title_authors_affiliations.pdf](https://track.editorialmanager.com/CL0/https:%2F%2Fjournals.plos.org%2Fplosone%2Fs%2Ffile%3Fid=ba62%2FPLOSOne_formatting_sample_title_authors_affiliations.pdf/1/010f0197f438383e-8fd3286f-ab0c-4e96-b630-e1a1da2f4836-000000/Khd_ip-gYGcyvQ0LYVrbuacwhBQjOMxNMtbP7HsIFZk=219)

Fulfilled

2. Thank you for stating the following in the Acknowledgments Section of your manuscript:

Please remove any funding-related text from the manuscript and let us know how you would like to update your Funding Statement. Currently, your Funding Statement reads as follows:

Funding statement has been removed from the acknowledgements section.

Please include your amended statements within your cover letter; we will change the online submission form on your behalf.

Updated funding statement provided below:

“This work was supported in part by grants from the New Frontiers in Research Fund (Exploration grant, CLP) and the Michael Smith Foundation for Health Research (Scholar Award, CLP). The funders had no role in study design, data collection and analysis, decision to publish, or preparation of the manuscript”

3. Thank you for stating the following financial disclosure:

““This work was supported in part by grants from the New Frontiers in Research Fund (Exploration grant, CLP) and the Michael Smith Foundation for Health Research (Scholar Award, CLP).””

Please state what role the funders took in the study.  If the funders had no role, please state: "The funders had no role in study design, data collection and analysis, decision to publish, or preparation of the manuscript."

Addressed, please refer to text in comment 2 above.

4. When completing the data availability statement of the submission form, you indicated that you will make your data available on acceptance. We strongly recommend all authors decide on a data sharing plan before acceptance, as the process can be lengthy and hold up publication timelines. Please note that, though access restrictions are acceptable now, your entire data will need to be made freely accessible if your manuscript is accepted for publication. This policy applies to all data except where public deposition would breach compliance with the protocol approved by your research ethics board. If you are unable to adhere to our open data policy, please kindly revise your statement to explain your reasoning and we will seek the editor's input on an exemption. Please be assured that, once you have provided your new statement, the assessment of your exemption will not hold up the peer review process.

Data has been made available at Borealis Dataverse Repository at the following DOI: https://doi.org/10.5683/SP3/SXVBGQ

5. Please include your full ethics statement in the ‘Methods’ section of your manuscript file. In your statement, please include the full name of the IRB or ethics committee who approved or waived your study, as well as whether or not you obtained informed written or verbal consent. If consent was waived for your study, please include this information in your statement as well.

Fulfilled; this can be found on Page 6 line 153-154, “Participants provided informed written consent before participation. This study was approved by the University of British Columbia Clinical Research Ethics Board.”

6. If the reviewer comments include a recommendation to cite specific previously published works, please review and evaluate these publications to determine whether they are relevant and should be cited. There is no requirement to cite these works unless the editor has indicated otherwise.

Not applicable

Reviewer #1

1. Ethics

Fulfilled

2. Inclusion/Exclusion Criteria:

Fulfilled

3. Flow Charts and Attrition

Figure created; Fig 4

4. Sex as a biological variable

Reported in Table 1

5. Subject Demographics

Reported in Table 1

6. Randomization

No randomization required; single group study

7. Blinding

Not applicable

8. Power Analysis

The following was added to the statistical Analysis section of the manuscript:

“Power for the mediation pathway was approximated by calculating power for each constituent effect (CB&M task performance→ physiological arousal; physiological arousal → RPS) using Cohen’s d and the pwr package, then combining them. Observed, standardized, and hypothetical medium-sized effects (d = 0.35) were evaluated for sensitivity.” (Page 11, Line 273-277)

9. Replication

Not required

10. Open Code

Not applicable

11. Open Data

The study data are publicly available in the Borealis Dataverse Repository at the following DOI: https://doi.org/10.5683/SP3/SXVBGQ

12. Self acknowledged Limitations

Limitations can be found in the Discussion session, Page 21, line 448; “Generalizability of the findings of our study are limited. Participants in the current study were in the chronic phase of recovery and had experienced most of the functional recovery expected post stroke [39], lived independently in the community, tended to have moderate level of lower extremity impairment, and reported medium to high level of self-reported balance confidence and low levels of state and trait anxiety. Therefore, future research should consider exploring these relationships in the sub-acute population when the post-stroke neural circuits are malleable and responsive to high intensity restorative treatments and perceptions of ability [40–42].”

13. Conflict of interest

Addressed on Page 22, line 475-476, “The authors declare no conflicts of interest.”

14. Funding Statement

Fulfilled

15. Registration Statement

Not Applicable

16. No Bar graphs of continuous data

Fulfilled

17. No rainbow color maps

Fulfilled

Reviewer #2

**Comment 1:** The authors need to do analyses including ABC, the subjective sense of balance in real life. In part since this showed a sex difference. This metric would seem, from first principle, to potentially have important implications for quality of life.

Response 1: The following has been added to the Discussion section of the manuscript to highlight the importance of studying this relationship in future studies.

“Specific to our use case of measurement of perception of stability while performing a walking task, further research is needed to address how changes in the physiological arousal response relates to balance self-efficacy over the course of rehabilitation of following stroke. The ABC, a measure of balance self-efficacy, has been shown to be a predictor of falls [39] and community reintegration [40] post-stroke . In this study, we were unable to examine ABC as a predictor of RPS and physiological arousal due to an insufficient sample size. This highlights an opportunity for future research exploring interactions between ABC scores, RPS ratings, and physiological arousal.” (Page 20, Lines 440-447)

**Comment 2:** The discussion of arousal is incomplete (e.g., l.395-). EDA is only one arousal measure, with heart rate variability, cortisol, and others also capturing arousal patterns that are missed with EDA. Thus, the authors should not overstate that arousal did not relate to RPS, since only one facet of arousal was measured.

Response 2:

In the introduction, we have described alternative methods for assessing physiological arousal and highlighted why EDA was the best measure of physiological arousal for our research question.

“Heart rate variability (HRV), cortisol production and electrodermal activity (EDA) can measure physiological arousal modulated by the autonomic nervous system (i.e., “fight or flight” response)[12,13]. EDA is a measurement of skin conductance driven exclusively by sympathetic nervous system activation [14], and is activated in conditions requiring heightened attention, state anxiety, or cognitive load [15]. EDA has shown to be a better metric to capture brief, task-related changes in sympathetic nervous system activity than metrics specific to HRV and cortisol production [16].” Page 4, Lines 101-107

We have also clarified (where applicable), that we are looking EDA as the physiological arousal measure.

“Contrary to our hypothesis physiological arousal, as measured by EDA, was not a mediator of the relationship between CB&M and RPS.” (Page 19, Line 416-417)

**Comment 3:** The abstract needs to be rewritten to be clearer, especially about what higher or lower in different measures indicate, and the interpretation of the relation between measures.
l.48 “repeat exposure:” does this mean first vs second test day? Is there precedent that that d1 and d2 of testing would be different?
l.57-58, 313- is unclear for “decreases in participant RPS scores relative of 2.80.” Does this mean that greater objective balance associates with reduced subjective balance?
l.63-64 what %variance explained by individual effects?

Response 3:

- Interpretation of results has been clarified.

“CB&M scores, indicating balance performance, explained 20.4% of the variability in the RPS. As CB&M task performance improved, RPS scores decreased by 2.80 (95% CI [-3.29 – -2.30]) to 3.67 (95% CI [-4.32 – -3.02]) points, indicating improved perceived stability.” (Page 2, Paragraph 3, line 57-60)

- Percentage of variance explained from individual effects has been clarified.
  “Participant-specific random effects accounted for more variance in the RPS than the fixed effects of task performance and physiological arousal, explaining 46% of variance in RPS.” (Page 2, Paragraph 3, line 63-64)

**Comment 4:** l.206 calculating baseline for EDR from 4 sec around the minimum could bias baseline measures, e.g. magnifying small dips from noise in the recording. While a reference is cited, this baseline measure requires clearer justification.

Response 4:

We ensured that the 4 second period of the signal was taken from the most stable portion of the signal. We have clarified this by adding the following into the Methods section.

“Manual inspection was employed to ensure the epoch representative of pre-task baseline was stable and did not include large fluctuations in the signal.” (Page 8, Paragraph 2, Line 209-210)

**Comment 5:** l.214 normalizing EDR to the maximum also needs good justification. Many studies use raw EDR, and normalizing could washout e.g. where people with larger EDR have less balance self-efficacy.

**Response 5:** Normalization was performed to enable comparisons across tasks between participants. The following was added to the manuscript to justify our approach.

“While larger EDA responses reflect greater autonomic arousal within individuals, it is difficult to compare the responses between subjects as baseline levels and response amplitudes can vary between people due to non-task related factors (e.g., medications, skin properties) [33]. Therefore, for each day, the maximum task-specific physiological arousal response out of all the tasks was identified for each participant separately. This participant-specific maximum response was used to normalize response values across all tasks performed by the individual to aid interpretation. Task-specific physiological arousal response values after normalization ranged from 0%-100%, with 100% representing the task that triggered the participant’s maximum response for that day. The normalized task-specific physiological arousal response for each item in the CB&M was used for data analyses. This preserved within-person response patterns while enabling group-level modeling of the relationship between EDA and perceived stability (RPS) across tasks.” (Page 9, Line 215-226)

**Comment 6:** l.253 are Tukey post hoc corrected for multiple comparisons?

**Response 6:** The following has been added to the Statistical Analysis section of the manuscript:

“Post hoc pairwise comparisons among CBM performance levels were conducted in R using the emmeans package with Tukey’s HSD adjustment, which corrects for multiple comparisons by controlling the family-wise error rate at α = 0.05.” (Page 11, Line 263-265)

**Comment 7:** l.184-185, 300- Please discuss more the concern is that removing measures in CBM will skew the remaining findings.

**Response 7:**

We have provided additional explanation for why some of the CB&M tasks were removed from analysis.

Methods:

“During data collection, some participants either declined to attempt tasks they perceived they would be unable to perform or performed tasks in a manner that did not adequately challenge their balance (i.e., use of physical assistance), leading to a CB&M score of 0. Therefore, CB&M items were only included in analyses if 1) at least 10 participants completed the task (to ensure enough observations were present) and 2) the mean CB&M score was at least 2 (indicating that on average, participants performed the task without physical assistance).” (Page 7, Line 184-189)

Results:

“Removing these tasks from analysis ensured that the corresponding physiological arousal response to a task was specifically attributable to balance being challenged.” (Page 15, Line 323-325)

**Comment 8:** l.137 change to “; and (3)”

**Response 8:** Addressed
