# Supplementary material for: Relationship between the Rate of Perceived Stability, electrodermal activity and task performance during balance challenges in chronic stroke
Source: PLoS One. 2026 Jan 8;21(1):e0322825. doi: 10.1371/journal.pone.0322825 (PMC12782383; doi:10.1371/journal.pone.0322825)
Supplement: Attachment — Submitted filename: Revisions Round 2_RPS EDA_Final.docx [file pone.0322825.s003.docx]

Dear Dr. Luca Citi,

We thank you and the reviewers for your thoughtful and constructive feedback of our manuscript entitled “Relationship between the Rate of Perceived Stability, electrodermal activity and task performance during balance challenges in chronic stroke”. We have carefully considered all comments and revised the manuscript accordingly.

**Editor Comments:**

**Comment #1:** Consider adjusting the paper title to reflect the relatively small sample size (“preliminary”?).

Response 1: We believe that the term “preliminary findings” may not accurately reflect the nature of this work. This manuscript presents the complete analysis from the full sample of 27 participants, rather than an early-phase or partial dataset. The findings are intended to inform future research rather than represent interim results. As suggested by the reviewers, we have noted that a larger sample size would enable the exploration of additional relationships among the variables examined in this study.

“Thus, future research inclusive of a larger sample size could explore lesion location as a potential moderator of the association between participant reported perception of stability and the physiological arousal response while performing tasks that challenge standing and walking balance.” Page # 22, Line 478-481

**Comment #2:** Discuss sex-related differences about risk factors, stroke subtypes, stroke severity, and outcome.

Response 2: We have added additional information regarding sex related differences.

“Sex differences have been previously reported in stroke subtypes, severity and recovery outcomes [21,22]. Although males have higher incidence of first-ever stroke compared to females [23], prognosis and recovery outcomes related to walking are reported to be poorer in females than males despite adjustment for baseline differences in age, pre-stroke function and comorbidities [22,24,25].” Page #5, Line # 128-132

**Comment #3**: Clarify the meaning of “repeat exposure”

Response #3: We have clarified the meaning of repeat exposure in both the abstract and the introduction.

Definition of repeated exposure in Abstract:

“This study aims to examine the relationships between EDA, task performance ability, and RPS in individuals with chronic stroke performing walking balance challenges. Further, the study explores how sex and repeat exposure (repeat performance of task) moderate these relationships.” Page #2, Line 43-46

Definition of repeated exposure in Introduction:

“This aim is addressed through three sequential objectives: 1) investigate the relationship between walking balance task performance scores and RPS ratings, and determine whether sex or repeated exposure (repeat performance of tasks) across days moderates this relationship” Page #5, Line #138-141

**Comment #4:** Provide additional details / clarify linear mixed model (exposure variable / fixed effects / random intercept / grouping structure)

Response #4: We have clarified the linear mixed model.

“Two different models were used for each objective, where the predictor (independent variable) was CB&M Task Performance for Objective 1 and physiological arousal for Objective 2. For both models (Model 1. CB&M task performance and Model 2. Physiological arousal for each CB&M task), each CB&M task was treated as a separate observation. Because multiple tasks were completed by each participant, the data had a hierarchical structure with tasks nested within participants. This grouping structure was modeled by including a random intercept for participant to account for within-subject correlation. Sex and repeated exposure (Day 1 vs Day 2) were explored as potential effect moderators by including them as separate interaction terms in both models, nested models were compared via likelihood ratio tests (p > 0.05 considered significant).  Tukey post hoc test was performed to assess pairwise differences between groups for all significant interaction terms.” Page #11, Line #268-278.

Comment #5: Briefly discuss future directions (potentially including lacunar vs non-lacunar ischemic strokes). Thoughts: discuss impact of type of lesion on perception of balance as a future area for study/exploration

Response #5: The following has been added to future lines of research;

“Furthermore, lesion location has shown to impact both balance and recovery of walking post stroke [48,49], and individuals with poorer walking and balance performance often report lower balance confidence, which is an independent predictor of perceived physical function, mobility, and recovery [5] .Thus future research inclusive of a larger sample size could explore lesion location as a potential moderator of the association between participant reported perception of stability and the physiological arousal response while performing tasks that challenge standing and walking balance.” Page #22, Line 475-481.

Comment #6: Check for additional typos, references, and minor cosmetic issues flagged in the reviewers' reports

Response #6: Manuscript has been revised for typos and cosmetic issues.

**REVIEWER #2:**

Now good. The paper provides new data, which are somewhat preliminary but help advance use of biometrics as indicators for risks like falls during stroke

Response to Reviewer #2: All comments addressed, no response required.

**REVIEWER #3**

The authors present the results of a potentially interesting revised version of an observational clinical study showing that ability to perform a walking balance task (CB&M task performance score) and the underlying physiological arousal response are independent predictors of perception of balance in people with chronic stroke as measured by the Rate of Perceived Stability. The study can improved if the following minor considerations are addressed:

**Comment #1:** Due to the small size of the study (n=27 patients) the title should clearly mention “preliminary findings”.

Response #1: Thank you for your comment and suggestion to clarify the scope of the study in the title. We believe that the term “preliminary findings” may not accurately reflect the nature of this work. This manuscript presents the complete analysis from the full sample of 27 participants, rather than an early-phase or partial dataset. The findings are intended to inform future research rather than represent interim results. As suggested by the reviewers, we have noted that a larger sample size would enable the exploration of additional relationships among the variables examined in this study.

“Furthermore, lesion location has shown to impact both balance and recovery of walking post stroke [48,49], and individuals with poorer walking and balance performance often report lower balance confidence, which is an independent predictor of perceived physical function, mobility, and recovery [5] .Thus, future research inclusive of a larger sample size could explore lesion location as a potential moderator of the association between participant reported perception of stability and the physiological arousal response while performing tasks that challenge standing and walking balance.” Page #22, Line 475-481.

**Comment #2:** It would be useful to clearly mention in the Introduction section that women differ from men in the distribution of risk factors and stroke subtypes, stroke severity, and outcome (see data and comment on the study published in Cerebrovasc Dis 2025;54(1):11-19. doi: 10.1159/000536436. Epub 2024 Jan 29. PMID: 38286114).

Response #2:

“Sex differences have been previously reported in stroke subtypes, severity and recovery outcomes [21,22]. Although males have higher incidence of first-ever stroke compared to females [23], prognosis and recovery outcomes related to walking are reported to be poorer in females than males despite adjustment for baseline differences in age, pre-stroke function and comorbidities [22,24,25].” Page #5, Line # 128-132

**Comment #3:**
It would be interesting to know the different stroke subtypes (cardioembolic stroke, lacunar infarct, infarct of unusual etiology, essential cerebral infarct, atherothrombotic infarct, intracerebral hemorrhage) in the study population.

Response #3:
“Inclusion criteria included: at least one year-since stroke (occurrence of stroke confirmed by medical records), hemiparesis post-stroke, the ability to walk at least 10 metres with or without a walking aid (confirmed during initial assessment)”. Page #7, Line #157-160

**Comment #4:** It would be interesting to add in the text that an essential line of future research would be precisely to evaluate the impact of the differences on this topic between lacunar and non-lacunar ischemic strokes. This recommendation is because the pathophysiology, prognosis, and clinical features of ischemic lacunar strokes are different from other acute ischemic cerebrovascular diseases (see and include this supporting reference: Neuroepidemiology 2010;35:231-236),

Response #4: The following has been added to the limitation section of the Discussion, and recommended references have been added.

“Furthermore, lesion location has shown to impact both balance and recovery of walking post stroke [48,49], and individuals with poorer walking and balance performance often report lower balance confidence, which is an independent predictor of perceived physical function, mobility, and recovery [5] .Thus, future research inclusive of a larger sample size could explore lesion location as a potential moderator of the association between participant reported perception of stability and the physiological arousal response while performing tasks that challenge standing and walking balance.” Page #22, Line 475-481.

**Comment #5:** A brief final comment on other possible lines of future research on the presented topic would be appreciated.

Response #5: This has been addressed partly in the comment above along with some changes in wording in the Discussion.

“Therefore, future research should consider exploring these relationships in the sub-acute population when the post-stroke neural circuits are malleable and responsive to high intensity restorative treatments and perceptions of ability [45–47]. Furthermore, lesion location has shown to impact both balance and recovery of walking post stroke [48,49], thus future research inclusive of a larger sample size could explore lesion location as a potential moderator of the association between participant reported perception of stability and the physiological arousal response while performing tasks that challenge standing and walking balance. This line of research has the potential to aid with interventions which aim to implement graded challenges to standing and walking balance during the active phase of stroke recovery. Furthermore, exploring these relationships specifically in people with chronic stroke living in the community with low balance confidence could provide insight into psychosocial presentations post stroke that leads to activity avoidance, sedentary behaviour and a lack of community reintegration associated with perception of walking balance [50].” Page #22, Lines 472-481

**Comment #6:** Please check references #26, #34 and #46

Response #6: Formatting for references have been fixed.

Reference #26 is now #29, Reference #34 is now #37, Reference #46 is now #51

**REVIEWER #4:**

**Comment #1:** Line 97 to 99 there is no figure but the figure caption is there
Response #1: Journal specific submission requirements state to not include the figure within the manuscript but to upload them as an attachment during the submission process.

**Comment #2:** Was participant subjected to walking 10 meters walk this before recruitment of it was base on self report?

Response #2:

“Inclusion criteria included: at least one year-since stroke (confirmed by medical records), hemiparesis post-stroke, the ability to walk at least 10 metres with or without a walking aid (confirmed during initial assessment).” Page #7, Line 157-160

**Comment #3**: Line 249-252 Move to the section where you explained the CB&M before this paragraph

Response #3: Has been moved to section where we talk about the CB&M. Page #8 Line 197-205

**Comment #4:** Linear Mixed Models line 253-261 is unclear, and I am having difficulty following exactly what was done. Please clearly specify the exposure variable(s) and the fixed effects included in your model. In addition, clarify how the random intercept was formulated and what grouping structure it represents.

Response #4: Section has been reworded to clarify the variables and effects.

“To address Objectives 1 and 2, Linear Mixed Models (LMMs) using the lmer function from the lme4 package [35]. Two different models were used for each objective, where the predictor (independent variable) was CB&M Task Performance for Objective 1 and physiological arousal for Objective 2. For both models (Model 1. CB&M task performance and Model 2. Physiological arousal for each CB&M task), each CB&M task was treated as a separate observation. Because multiple tasks were completed by each participant, the data had a hierarchical structure with tasks nested within participants. This grouping structure was modeled by including a random intercept for participant to account for within-subject correlation. Sex and repeated exposure (Day 1 vs Day 2) were explored as potential effect moderators by including them as separate interaction terms in both models, nested models were compared via likelihood ratio tests (p > 0.05 considered significant).  Tukey post hoc test was performed to assess pairwise differences between groups for all significant interaction terms.” Page #11, Line #267-278.

**Comment #5**: “Power for the mediation pathway was approximated by calculating power for each constituent effect (CB&M task performance→ physiological arousal; physiological arousal → RPS) using Cohen’s d and the pwr package, then combining them. Observed, standardized, and hypothetical medium-sized effects (d = 0.35) were evaluated for sensitivity." -The approach described here approximates power by calculating it separately for each constituent effect and then combining them. This should be noted as a limitation, because the true power for the mediation pathway is typically lower, both paths must be significant for the indirect effect to hold. As a result, this approach may overestimate the true mediation power.

Response #5: Further explanation is provided;

“However, power for the mediation analysis was approximated by calculating it separately for each constituent path and then combining them. This approach, while practical, may overestimate the true power for detecting the mediation effect. Consequently, the mediation analysis should be considered exploratory.” Page #21, Line 443-447

**REVIEWER #5:**

**Comment #1**: l.48 “repeat exposure:” does this mean first vs second test day? Is there precedent that that d1 and d2 of testing would be different?

Response #1: The definition of repeat exposure and precedent has been clarified in both the abstract and the introduction.

Abstract: “Measurement of electrodermal activation (EDA), modulated by the autonomic nervous system, during perturbations to standing balance reflects the physiological arousal (‘fight or flight’) response of the individual as they maintain or recover their balance. Repeat performance of a balance task has been shown to result in habituation of EDA within a single session; however, studies have yet to test whether similar habituation occurs when the same balance tasks are repeated across different days.” Page 2, Line 38-43

Introduction: “Furthermore, as clinical walking balance interventions extend over multiple sessions, it would be important to determine whether these relationships change with repeated exposure to the walking challenges on subsequent days to enhance the clinical utility and interpretability of treatment intensity measures. Repeat performance of a balance task has been shown to result in habituation of EDA within a single session [18]; however, studies have yet to test whether similar habituation occurs when the same balance tasks are repeated across different days.” Page #5, Line #121-127
